# An open label randomized clinical trial comparing the safety and effectiveness of one, two or three weekly pentamidine isethionate doses (seven milligrams per kilogram) in the treatment of cutaneous leishmaniasis in the Amazon Region

**DOI:** 10.1371/journal.pntd.0006850

**Published:** 2018-10-31

**Authors:** Ellen Priscilla Nunes Gadelha, Rajendranath Ramasawmy, Bruna da Costa Oliveira, Nágila Morais Rocha, Jorge Augusto de Oliveira Guerra, George Allan Villa Rouco da Silva, Tirza Gabrielle Ramos de Mesquita, Carolina Chrusciak Talhari Cortez, Anette Chrusciak Talhari

**Affiliations:** 1 Tropical Medicine Post-graduation Program. Heitor Vieira Dourado Amazon Tropical Medicine Foundation and Amazonas State University, Manaus, AM, Brazil; 2 Department of Molecular Biology, Division of Immunogenetics, at the Tropical Medicine Foundation—Dr Heitor Vieira Dourado and Universidade Nilton Lins, Manaus, Amazonas, Brazil; 3 Department of Leishmaniasis, Research Division at the Tropical Medicine Foundation—Dr Heitor Vieira Dourado, Manaus, Amazonas, Brazil; 4 Department of Dermatology. Heitor Vieira Dourado Amazon Tropical Medicine Foundation, Manaus, AM, Brazil; National Institute of Allergy and Infectious Diseases, UNITED STATES

## Abstract

**Background:**

American Cutaneous Leishmaniasis (ACL), a vector borne disease, is caused by various species of *Leishmania* and in the Amazonas, *Leishmania guyanensis* is predominant. The recommended drugs for treatment of cutaneous leishmaniasis (CL) in Brazil are pentavalent antimonials, pentamidine isethionate (PI) and amphotericin B. Pentamidine was initially used as metanolsulfonate or mesylate (Lomidine) at a dose of 4 mg/kg/daily, containing 2.3mg of base. This drug was withdrawn from the market in the eighties, and currently is available as PI. The PI dose required to achieve an equivalent dose of pentamidine base is 7 mg/kg, rather than the 4 mg/kg that is currently recommended in Brazil.

**Objectives:**

The aim of this study was to evaluate the efficacy and safety of PI in a single dose, two or three doses of 7 mg/kg body weight, intramuscularly, with an interval of seven days between each dose.

**Materials and methods:**

This study was conducted as a controlled, randomized, open–label clinical trial for a total number of 159 patients with CL. Individuals aged 16–64 years with one to six lesions of confirmed CL based on amastigotes visualization in direct examination of Giemsa stained of dermal scraping from the border of the lesion with no previous treatment for CL and no abnormal values for liver enzymes were eligible to participate in the study. Patients with history of diabetes, cardiac, renal, and hepatic disease as well as pregnant women were excluded. Cure was defined as complete healing in the diameters of the ulcers and lesions skin six months after the end of the treatment.

**Results:**

From November 2013 to December 2015, 159 patients were screened and allocated in three groups for treatment with PI: i) 53 patients were treated with a single dose intramuscularly injection of 7 mg/kg body weight; ii) 53 received two doses of 7 mg/kg within an interval of seven days; and iii) 53 were treated with three doses of 7mg/kg with an interval of seven days between each dose. In 120 patients, *L*. *guyanensis* was identified. A cure rate of 45%, 81.1% and 96.2% were observed in the first, second and third group, respectively. The cure in the three PI dose group was higher compared to the single-dose (p<0.0001) and two-dose groups (p = 0.03). No serious adverse events occurred.

**Conclusion:**

The present study shows that PI is a safe drug and its efficacy varied with the number of doses. The administration of PI in patients with ACL, predominantly caused by *L*. *guyanensis*, was mostly efficient in three or two doses of 7 mg/kg.

**Trial registration:**

ClinicalTrials.gov NCT02919605

## Author summary

Ninety percent of all cases of CL are concentrated in five countries, including Brazil. Brazil is among the most endemic countries in the Americas. According to the Brazilian Ministry of Health, 30,000 new cases are diagnosed every year and the prevalent species are *L*. *braziliensis* and *L*. *guyanensis*. In the region of Manaus (Western Amazon), *L*. *guyanensis* is responsible for 95% of the cases of CL. The recommended drugs for treatment of CL in Brazil are pentavalent antimonials, PI and amphotericin B (AmB). Pentavalent antimonial is the first-line treatment in Brazil, and AmB is recommended as second-line. The recommended dose for the treatment of CL is 10–20 mg/kg/day of meglumine antimoniate for a period of 20 days. Its efficacy varies from 26.3% to 81.6%. For treatment with AmB, the patient must come to the hospital and be monitored for renal function for several hours. We report a phase II pilot study comprising 159 patients with CL due to *L*. *guyanensis* treated with PI. The patients were randomly divided in three groups: 53 received a single intramuscular injection of 7mg/kg PI salt; 53 received a regimen of two intramuscular injections of 7 mg/kg within a seven-day interval; and 53 were treated with three intramuscular injections of 7 mg/kg with a seven-day interval between each dose. All patients live in the region of Manaus and were monitored at Tropical Medicine Foundation of Amazonas. Patients underwent a dermatological and laboratorial examination at the start of the study and at weeks 1 and 4. PI was well tolerated and no severe adverse effects were detected. After a 6-month follow-up, we observed 81.% and 96.2% effectiveness for the cure rates in the groups receiving two and three intramuscular injections of 7 mg/kg PI in ACL patients at one-week intervals between doses, respectively. Only 45.3% of the patients treated with a single dose of the medication were considered to be cured. The use of PI in the aforementioned doses is the best option to treat ACL in this region given the long distance, the difficulty in accessing health centers, and the *L*. *(V*.*) guyanensis* predominance in the Brazilian Amazon. We recommend either two or three weekly doses of PI at 7 mg/kg for the treatment of ACL patients in the region.

## Introduction

American cutaneous leishmaniasis (ACL) is an infectious vector-borne disease caused by different protozoan parasites belonging to the genus *Leishmania*. The disease was regarded mainly as an occupational disease affecting people working in tropical forested areas, where they are exposed to the natural transmission cycle of the disease. Changes in these environments lead to the proliferation of various species of the vector, their associated parasites, and reservoirs around rural settlements. The presence of vectors and infection in peri-urban zones, that were not previously endemic areas, are emerging. [1,2]

In Brazil, the most important species are *Leishmania (Viannia) braziliensis*, which is found throughout the country and *Leishmania (Viannia) guyanensis*, which affects mainly the northern part of the country especially the state of Amazonas. [3,4,5,6,7,8]*Leishmania (Leishmania) amazonensis*. *Leishmania (Viannia) lainsoni*, *Leishmania (Viannia) naiffi*, *Lieshmania (Viannia) shawi*, and *Leishmania (Viannia) lindenbergi* are also present.[9]

ACL may present with cutaneous and/or mucosal involvement. Clinically, cutaneous leishmaniasis (CL) may manifest as a painless single or multiple deep ulcerated skin lesions affecting any exposed parts of the body. The lesions can also show a verrucous, vegetative aspect or appear as papules, nodules, and infiltrative lesions. Mucosal involvement is rarely seen in ACL caused by *L*. *(V*.*) guyanensis*. [10,11,12,13]

The worldwide prevalence of leishmaniasis, including the visceral form of the disease, is about 12 million. According to the World Health Organization (WHO), 350 million people are considered to be at risk. About 90% of all cases of CL are concentrated in five countries and Brazil is one of the most endemic countries in the Americas.[14] In 2015, 19,395 cases of ACL were diagnosed in Brazil and 1,713 of those cases were from the state of Amazonas.[15] 47.5% of the 1,713 cases were diagnosed and treated at the Tropical Medicine Foundation Dr. Heitor Vieira Dourado (FMT-HVD), a reference center for infectious disease in Manaus, capital of the Amazonas state.

In Brazil, meglumine antimoniate is the drug of choice for the treatment of ACL; amphotericin B (AmB) and pentamidine are the second-line therapeutic options.[1] Recently, Miltefosine was shown to be effective and safe for the treatment of ACL caused by *L*. *guyanensis* and *L*. *braziliensis*, but it is not yet available in the country.[8,9] At the recommended dose of 10–20 mg/kg/day injected for 20 days[1], the first-line therapy, meglumine antimoniate has efficacy rates ranging from 26.3% to 81.6%.[16,17] The second-line therapy, AmB is limited by the requirement for hospital administration and laboratory monitoring of renal function which poses logistical and financial challenges.[18]

Pentamidine is an aromatic diamidine with a relevant anti-parasitic activity. Since it was first synthesized in the early 1940s, it has been widely used for treating human African trypanosomiasis, infection by *Pneumocystis jirovecii*, and visceral and CL.[19,20] Pentamidine mesilate (Lomidine) was withdrawn from the market in the 1980s, and only pentamidine isethionate (PI) (Pentacarinat, Pentam) is currently available. Pentamidine was initially used as metanolsulfonate or mesylate (Lomidine) at a dose of 4 mg/kg/daily, containing 2.3mg of base. The PI dose required to achieve an equivalent dose of pentamidine base is 7 mg/kg, rather than the 4 mg/kg that is currently recommended in Brazil.[21] Nevertheless, the Brazilian Ministry of health (BMH) guidelines still recommends a three-day regimen of 4mg/kg per injection with a maximum dosage of 2 g.

The present study aims to evaluate the efficacy and safety of one, two, or three intramuscularly injections of 7 mg/kg PI at seven day interval for the treatment of ACL in a population with predominantly *L*. *guyanensis* infection.

## Materials and methods

### Ethics statement

The study was approved by the Research and Ethics Committee of the FMT-HVD. Written informed consent was obtained from the patients enrolled in the study. For patients under 18 years old, written informed consent was obtained from parents or legal guardians. The study was registered at ClinicalTrials.gov (NCT02919605).

### Study design

We conducted an open-label, randomized, and controlled phase-II clinical trial from November 2013 to December 2015 at the outpatient clinic of the Service of Dermatology at FMT-HVD in Manaus, Amazonas, Brazil. Two hundred and fifty patients with parasitological confirmed diagnosis of CL were recruited at the FMT-HVD. CL was defined as the presence of up to six ulcerous lesions with no lymphatic or mucosal diseases and amastigotes visualized in direct examination of Giemsa stained of dermal scraping from the border of the lesion.

### Inclusion and exclusion criteria

Individuals aged 16–64 years with one to six lesions of confirmed CL based on case definition with no previous treatment for CL were eligible to participate in the study.

The exclusion criteria were patients with CL treated in the previous three months; protein–calorie malnutrition; pregnancy or lactation; inability to attend one of the study visits; medical history of diabetes mellitus; cardiac, renal, and hepatic disease; and abnormal baseline values for amylase, creatine phosphokinase (CPK), alkaline phosphatase (ALP), aspartate aminotransferase (ALT), alanine aminotransferase (AST), creatinine, and glucose.

**Fig 1:**
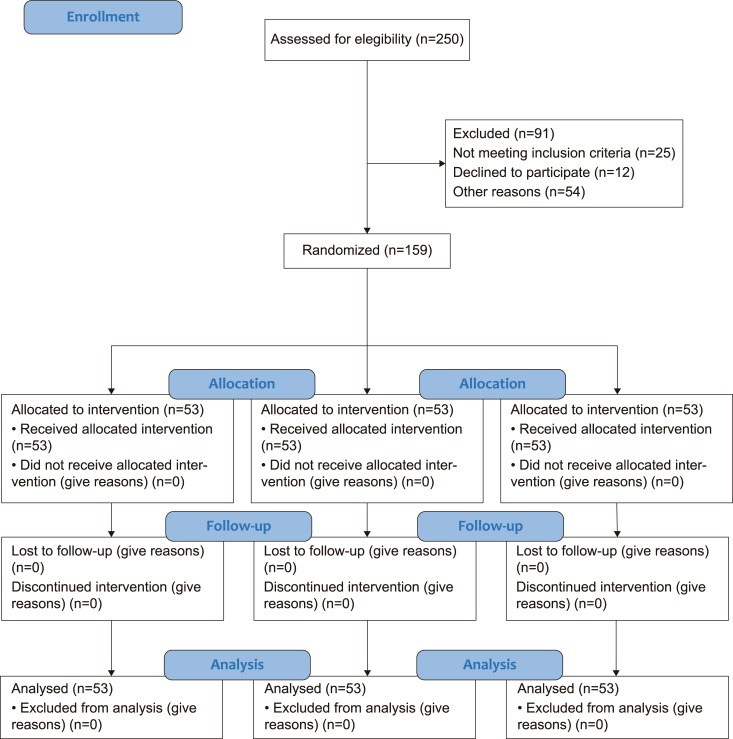
Flow Diagram of the progress through the phases: enrolment, intervention, allocation, follow-up and analysis.

The flow diagram of participants is described in Fig 1.

### Sample size

The sample size was calculated by using the difference between proportions test by considering the alpha and beta errors. To achieve statistical significance, 53 individuals were sufficient for each group. The cure rate estimated for the group treated with three PI doses was 80%, and that for the group treated with a single PI dose was 58.1% at a power of 80% and a confidence level of 95%.

### Randomization and masking

Randomization was performed by a statistician with no clinical involvement in the trial using a random allocation sequence generated by the open software available at www.randomization.com. Eligible subjects were randomly allocated to receive one of the following regimens in a 1:1:1 ratio: one, two, or three intramuscularly injections of 7 mg/kg PI within a seven-day interval.

The allocation sequence was concealed in sequentially numbered, sealed envelopes until interventions were assigned. Patients chose one envelope and accordingly is assigned to one of the group. Injections were administered by a nurse aware of the intervention allocation. Treatment assignment could not be masked to subjects due to the intramuscular injections.

### Study procedures

After written informed consent was obtained from the eligible individuals, a detailed dermatological examination of the lesion(s) was performed to identify its location and number as well as the presence or absence of clinical local regional lymphadenitis. The lesions were photographed and measured. The size of the lesion was defined as the diameter of the largest lesion measured in millimeters. Clinical evaluation was conducted at enrollment, during the treatment visits, and during the follow-up visits 1, 4, 8, and 24 weeks after treatment.

Clinical and laboratory adverse events were graded according to the Common Terminology Criteria for Adverse Events (CTCAE version 5.0) of the National Cancer Institute (http://ctep.cancer.gov/reporting/ctc.html). CTCAE consider grade 1: asymptomatic or mild; grade 2: moderate, non-invasive medical intervention indicated; grade 3: severe or medically significant but not immediately life-threatening; grade 4: life-threatening; grade 5: death.

Screening laboratory tests consisting of complete blood count, stool examination, urine test, rapid HIV test, urea, creatinine, ALP, ALT, AST, CPK, amylase, glucose, and beta HCG test for women in child-bearing age were performed prior to inclusion. One skin biopsy of 4 mm was taken from the largest lesion for parasitological diagnosis through the microscopic examination of biopsy smears and histopathology.

### Identification of Leishmania spp. by direct nucleotide sequencing

DNA was either prepared from lesion biopsy specimens of all the patients with CL or from parasite’s culture. Briefly, promastigotes were cultured in blood agar medium of Novy and McNeal modified by Nicolle (NNN) and Schneider. Polymerase chain reaction was performed to amplify a fragment of the Hsp 70 gene and of miniexon of *Leishmania* sp. The following pair of primers: HSP70F:5’-GGACGAGATCGAGCGCATGGT-3’ and HSP70R: 5’-TCCTTCGACGCTCCTGGTTG-3’ for HSP70 and Mini-ExonF:5’-TATTGGTATGCGAAACTTCCG-3’ and Mini-ExonR: 5’-ACAGAAAACTGATACTTATATAGCG-3’ were used to amplify separately a fragment of 233bp for HSP70 and 227 bp for Mini-Exon respectively. PCR amplicons were precipitated with polyethylene glycol. The purified amplicons were sequenced using the BigDyeTerminator Kit (3.1) (Life technologies) according to the protocol suggested by the manufacturer. Both sense and antisense primers used in PCR of each gene were also used for sequencing reaction separately. The sequencing product was purified with Ethanol / EDTA / Sodium Acetate, according to the recommendations of Life Technologies and submitted to capillary electrophoresis in the ABI 3130xL Genetic Analyzer (Applied Biosystems) and the resulting electropherograms were edited and analyzed using the Sequencing Analysis Program (Life technologies, version 5.3.1) and nucleotide blast using the site https://blast.ncbi.nlm.nih.gov/Blast.cgi?PROGRAM to discriminate the species.

### Drug administration

The patients were randomly assigned to one of the three groups: 53 received a single intramuscular injection of 7mg/kg PI salt; 53 received a regimen of two intramuscular injections of 7 mg/kg within a seven-day interval; and 53 were treated with three intramuscular injections of 7 mg/kg with a seven-day interval between each dose. The same lot of PI (Pentacarinat, Sanofi-Aventis, Anagni, Italy), number 1A5019, was used in all of the subjects, provided by the BMH. The total dose of PI (maximum of 1,95 mg) was divided in two, and each half was applied to each gluteal zone. This practical experience, which was acquired in a previous clinical trial[10}, decreases the likelihood of nodule and abscess formation. PI injections were administered at the FMT-HVD Day Hospital, where the patients remained at rest for 1 h. All patients were instructed to eat carbohydrate-rich food before receiving the PI injection. Capillary blood glucose was measured 30 min before and after the procedure.

### Clinical endpoint criteria

Number of patients with complete healing in the diameters of the ulcers and lesions skin six months after the end of the treatment was defined as the primary outcome, while a 50% reduction in lesion diameters, two months after the end of the treatment was considered as the secondary outcome. Clinical failure was defined as the emergence of new lesions or a 50% increase in previously documented lesions eight weeks after the treatment was concluded. Rescue therapy for clinical failure was the administration of 20 mg/Sb (meglumine antimoniate)/kg body weight per day for 20 days according to the BMH recommendation.[1]

### Statistical analysis

Data were analyzed using the Statistical Package for the Social Sciences version 16.0. and the R 3.2.2 software (*The package used is in the link*: *https*: *https*:*//stat*.*ethz*.*ch/R-manual/R-devel/library/stats/html/fisher*.*test*.*html**)* Initial descriptive studies were performed through frequency tables, position measurements, and variability. Fisher’s exact test was used to analyze the categorical variables and healing rates, and the non-parametric Kruskal–Wallis test was used to compare the means (quantitative variables). The significance level was 0.05, and the confidence level was 95%. Subgroup and Post hoc analyses were performed in relation to variables of sex and age with the outcome of the patient.

## Results

In total, 250 patients presenting with ACL and positive skin smear for *Leishmania* were assessed as shown in the flowchart (Fig 1). Ninety-one patients were excluded for the following reasons: living far from Manaus (n = 41), unavailability for visits (n = 8), refusing to participate in the study (n = 12), lesion presenting more than a 5 cm diameter (n = 8), previous treatments (n = 15), and associated chronic diseases (n = 7). All of the included patients (n = 159) completed the treatment follow-up, which was set for six months (Table 1), irrespective of the doses applied.

**Table 1:** Baseline characteristics of the included patients with ACL in Manaus in 2013–2015.

Characteristics	one dose	two doses	three doses
Age in years (range)			
	***p = 0*.*899***		
< 18	1 (1,9)	1 (1,9)	2 (3,8)
18 |— 36	30 (56,6)	21 (50,9)	29 (54,7)
36 |— 54	20 (37,7)	17 (37,7)	20 (37,7)
> = 54	2 (3,8)	4 (9,4)	2 (3,8)
Genre			
	***p = 0*.*134***		
Female	17 (32,1)	12 (22,6)	8 (15,1)
Male	36 (67,9)	41 (77,4)	45 (84,9
			
No. of lesions (%)	***p = 0*.*024***		
1	33 (62,3)	26 (49,1)	25 (47,2)
2	11 (20,8)	10 (18,9)	13 (24,5)
3	8 (15,1)	6 (11,3)	8 (15,1)
4	1 (1,9)	8 (15,1)	1 (1,9)
5		3 (5,7)	2 (3,8)
6			4 (7,5)
Fisher's Exact Test			

Overall, 122 (76.7%) patients were males and 37 (23.3%) were females. The average age was 32 years old. Eighty-four patients had a single lesion, 34 had two lesions, 22 had three lesions, 10 had four lesions, 5 had five lesions, and 4 had six lesions. Most of the lesions were located in the upper limbs (Table 1).

The etiologic agent was identified in 120 cases and distributed as follows: *L*. *guyanensis* (114 patients), *L*. *naifi* (4 patients), and *L*. *braziliensis* (2 patients). The diagnosis of the 39 remaining patients was confirmed through positive skin smear without species identification.

Some patients presented with clinical manifestations, such as papules adjacent to the lesion (n = 10), local regional lymphadenitis (n = 36), and secondary bacterial infection (n = 7), before treatment. These events did not seem to be related to the clinical outcome of the treatment.

Treatment effectiveness was evaluated in all of the 159 patients with ACL regardless of the identified *Leishmania* species. The clinically cured proportion was 45.3% (24/53) in the single PI dose group, 81.1% (43/53) in the two PI dose group, and 96.2% (51/53) in the three PI dose group. The cure in the three PI dose group was higher compared to the single-dose (Fisher exact test p<0.0001) and two-dose groups (Fisher exact test p = 0.03).

Among the 29 patients classified as clinical failures in the single dose group, three (10.3%) never healed the leishmania lesions and 26 (89.6%) presented complete epithelialization but relapsed afterwards (apparent cure with recidiva cutis). In the two-doses group, one (10%) out of the 10 clinical failures patients never healed and 9 (90%) presented complete epithelialization but also relapsed afterwards. In the three-doses group only two patients presented complete epithelialization but relapsed afterward. Primary and secondary outcomes efficacy are displayed in Table 2.

**Table 2:** Follow-up endpoint results in treatment groups.

Follow-up endpoints	one dose		two doses		three doses		p-valor
	healed	failed	healed	failed	healed	failed	
2 months after treatment							
No. of patients healed/failed	50	3	52	1	53	0	0,325
%	94,3%	5,7%	98,1%	1,9%	100,0%	0,0%	
confidence interval 95%	(83,3–98,5)		(88,6–99,99)		-		
6 months after treatment							
No. of patients healed/failed	24	29	43	10	51	2	<0,001
%	45,3%	54,7%	81,1%	18,9%	96,2%	3,8%	
confidence interval 95%	(33,5–61,2)		(67,6–90,1)		(85,9–99,3)		
Fisher's Exact Test							

The analyses of sex, age, number and topography of the lesions did not show any statistical significance between treatment groups (Table 3).

**Table 3:** Responses to treatment at follow-up six months after the treatment for ACL according to sex and age of patients in Manaus in 2013–2015.

Characteristics	Single PI dose			2 PI doses			3 PI doses			Total
	Cure	%	Total	Cure	%	Total	Cure	%	Total	
**Sex**	** **	** **	** **	** **	** **	** **	** **	** **	** **	** **
Female	9	*52*,*9*	*17*	9	*75*,*0*	*12*	7	*87*,*5*	*8*	37
Male	15	*41*,*7*	*36*	34	*82*,*9*	*41*	44	*97*,*8*	*45*	122
	*p = 0*,*558*			*p = 0*,*677*			*p = 0*,*282*			** **
**Age**										
< 18	1	*100*,*0*	1	1	*100*,*0*	1	2	*100*,*0*	2	4
18–36	17	*56*,*7*	30	21	*77*,*8*	27	28	*96*,*6*	29	86
36–54	6	*30*,*0*	20	17	*85*,*0*	20	19	*95*,*0*	20	60
> 54	0	*0*,*0*	2	4	*80*,*0*	5	2	*100*,*0*	2	9
	*p = 0*,*057*			*p = 0*,*899*			*p >0*,*99*			

*significant for Fisher exact test

Overall, PI was well tolerated by the patients. No serious adverse events (SAE) occurred and none of the reported adverse events (AE) required discontinuation of therapy in any patient. Some patients presented with erythema and swelling at the injection site. Asthenia, fever, malaise, and headache were also reported. As expected, these adverse events were more often reported by patients treated with three PI doses than by those treated with one or two doses (Table 4). Pain was the most frequent AE, 128 patients experienced grade 1 and 8 Patients grade 2. Twenty-three patients reported no AE. A 54-year-old male patient with a family history of diabetes developed type 2 diabetes mellitus one month after the treatment was concluded. This patient was treated with three PI doses with 1,764 mg PI.

**Table 4:** Adverse events six months after treatment according to the treatment group of ACL patients in Manaus in 2013–2015.

Adverse events	Treatment						Total	p-value
	Single dose		2 PI doses		3 PI doses			
	N	*% (n/53)*	N	*% (n/53)*	N	*% (n/53)*		
**Pain**	41	*77*,*4*	47	*88*,*7*	48	*90*,*6*	136	0,135
**Erythema**	11	*20*,*8*	21	*39*,*6*	18	*34*,*0*	50	0,099
**Swelling at injection site**	15	*28*,*3*	17	*32*,*1*	16	*30*,*2*	48	0,701
**Astenia**	6	*11*,*3*	13	*24*,*5*	16	*30*,*2*	35	*0*,*054*
**Local pruritus**	1	*1*,*9*	1	*1*,*9*	6	*11*,*3*	8	*0*,*051*
**Fever**	3	*5*,*7*	4	*7*,*5*	2	*3*,*8*	9	0,909
**Malaise**	6	*11*,*3*	10	*18*,*9*	12	*22*,*6*	28	0,347
**Headache**	2	*3*,*8*	0	*0*,*0*	6	*11*,*3*	8	*0*,029*
**Taste change**	1	*1*,*9*	2	*3*,*8*	2	*3*,*8*	5	0,999
**Abscess**	5	*9*,*4*	4	*7*,*5*	6	*11*,*3*	15	0,942
**Nausea**	0	*0*,*0*	1	*1*,*9*	1	*1*,*9*	2	0,999
**Shortness of breath after injection**	1	*1*,*9*	1	*1*,*9*	0	*0*,*0*	2	0,999
**Vomiting**	0	*0*,*0*	0	*0*,*0*	1	*1*,*9*	1	0,999
**Paresthesia**	0	*0*,*0*	0	*0*,*0*	1	*1*,*9*	1	0,999
**Vaginal bleeding**	0	*0*,*0*	1	*1*,*9*	0	*0*,*0*	1	0,999
**Urticaria**	1	*1*,*9*	1	*1*,*9*	2	*3*,*8*	4	0,999

* significant for Fisher exact test

**Fig 2:**
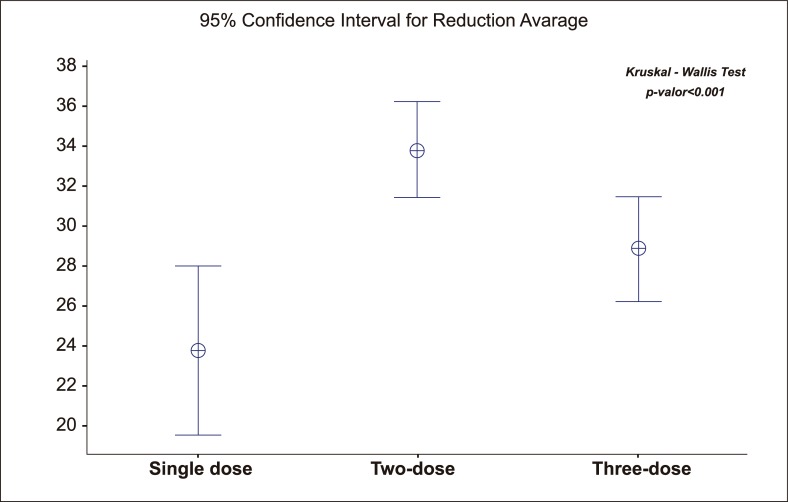
Average of the capillary glycemia reduction before and after half an hour of the applications between the treatments.

Leukocytosis and discrete CPK, ALP, urea, and creatinine increase were observed one week after the treatment in all the patients. These values returned to normal one month after the treatment. The blood glucose level, measured 30 minutes before and after the injections, showed a significant reduction in groups treated with two and three PI doses (Fig 2).

## Discussion

Currently, the BMH recommends that the medication for patients should consider clinical form and species of *Leishmania*. As there are no specific routine examinations in the health services to identify the infecting species, the recommendations are based on the preexisting evidence on the circulation of the parasite species of the endemic area. Treatment decisions are made with consideration for the local epidemiology, and patient clinical features. According to the BMH, it is common to have therapeutic failures or incomplete healing of CL. It is important to follow patients for up to six months after the end of treatment.[1]

The state of Amazonas is among the first Brazilian states to adopt PI as first-line treatment for ACL since 1985.[11] The first clinical trials, which applied the doses recommended by the BMH, showed PI effectiveness similar, or superior, to that of antimonials even in patients presenting with mucosal lesions.[18]

Therapeutic failures associated with the use of antimony and PI have been reported, and they have considerably increased in recent years.[8,11,19] Several hypotheses may explain the decreased effectiveness of anti-*Leishmania* drugs. The adoption of low PI doses in the northern region and patients’ genetic variability are among them. The presence of the double-stranded RNA virus (Leishmania RNA virus 1) has also been suggested to increase the risk of failure in first-line treatments.[22]

In recent years, several clinical trials showed the actual efficacy of PI for CL.[8,11,19] In 2015, a non-inferiority trial conducted in Suriname where *L*. *guyanensis* is predominant concluded that the three-day regimen (two injections of 7 mg/kg PI in three days) was non-inferior to the seven-day standard regimen (three injections of 4 mg/kg PI in seven days) in terms of clinical and parasitological cure.[23]

In the Amazon Region, the regimen efficacy of PI, as recommended by the BMH, is 58.1% for the treatment of CL caused by *L*. *guyanensis*.[18] Recently, our group showed an efficacy of 55% with a single dosage of 7 mg/kg PI for the treatment of CL due to *L*. *guyanensis*.[24] These results are similar to those in the standard regimen recommended by the BMH.[18]

The findings of this study are particularly important, as a study conducted at FMT-HVD in 2011 recorded a reduced effectiveness of antimonials (55.5%), the first-line therapeutic approach for ACL.[18] Of note, treatment with PI needs only one, two or three injections in contrast to the use of antimony where there is one intramuscular injection per day for 20 days and this leads to treatment abandon by many patients.

One of the main issues related to PI use is administering it through the deep intramuscular route, as its superficial administration may lead to local reactions such as nodule formation, fistulization, and ulceration. Therefore, the medication is recommended to be administered inside the outpatient clinic by a trained technician. However, compared with a treatment that requires at least 20 antimonial injections or one that requires monitoring the patient for several hours to administer amphotericin B, PI administration can be inferred to be less complex and less expensive.

Another key aspect related to PI is the need for patients to be well fed prior to the treatment. Hypoglycemia and lipothymic reactions are often recorded among patients who do not receive proper instructions. Diabetes was commonly reported in African patients, mainly those in Ethiopia, who were treated with a daily series of 15–20 pentamidine mesylate injections at total doses higher than 3–4 g.[25] One out of the 159 patients treated with PI in the present study developed diabetes. The patient had a family history of diabetes, received a total PI dose of 1.76 g. Some patients reported general symptoms, such as asthenia, fever, malaise, headache, taste change, and nausea. Transient biochemical changes were also observed.

One of the limitations of this study is that we were not able to identify the causative *Leishmania* species affecting all our participants. In 39 out of the 159 patients, PCR for *Leishmania* was not successful. However, in this region most of the studies report a 95% infection with *L*. *guyanensis*.[2]. Another limitation is the non-blind evaluation by the medical staff, which may cause a bias in the recording of data. However, we believed that the medical staff is well trained for carrying clinical trials and followed the codes of ethics. Lastly, the single dose group was evaluated 2 weeks earlier than the two or three doses groups.

## Conclusion

Altogether, we showed that the administering of two or three doses of PI at 7mg/kg to ACL patients infected by *L*. *guyanensis*, have a better cure in comparison with one dose. All dosing regimens showed adequate safety. It will be interesting to test the same dose in other regions of endemicity of CL caused by other *Leishmania* sp. We recommend two or three weekly doses of PI at 7 mg/kg for the treatment of ACL patients.

## Supporting information

S1 Consort Checklist (DOC)

S1 Trial Protocol (TIF)
