# Supplementary figures and images for: An open label randomized clinical trial comparing the safety and effectiveness of one, two or three weekly pentamidine isethionate doses (seven milligrams per kilogram) in the treatment of cutaneous leishmaniasis in the Amazon Region
Source: PLoS Negl Trop Dis. 2018 Oct 31;12(10):e0006850. doi: 10.1371/journal.pntd.0006850 (PMC6231690; doi:10.1371/journal.pntd.0006850)

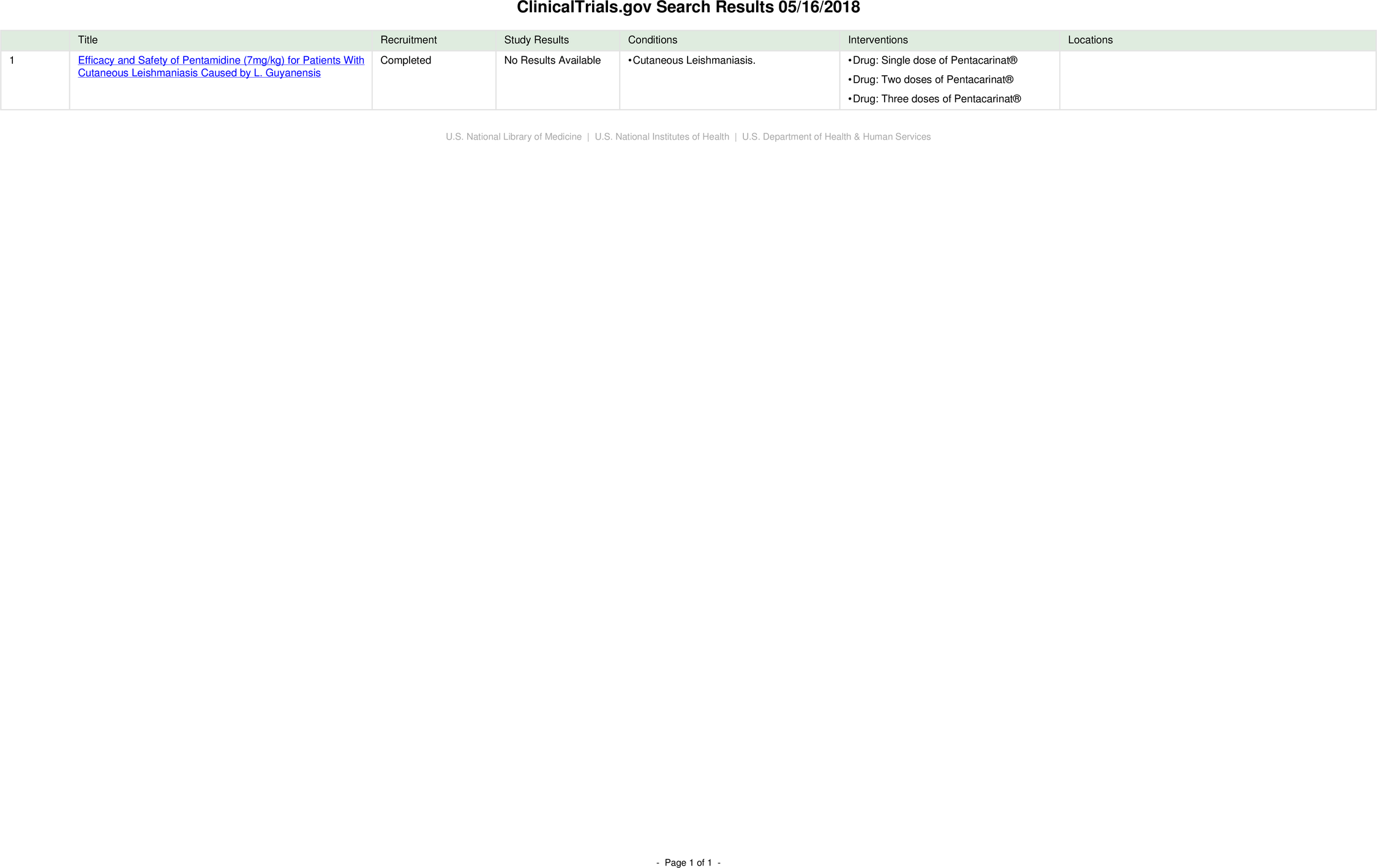

Supplement: S1 Trial Protocol — (TIF) [file pntd.0006850.s002.tif]
